# Long-term follow-up of S0221, comparing alternative dose-schedules of anthracycline and taxane therapy in early breast cancer

**DOI:** 10.1093/jncics/pkag024

**Published:** 2026-03-15

**Authors:** Azka Ali, William E Barlow, Halle C F Moore, Timothy J Hobday, Claudine Isaacs, Muhammad Salim, Kathy S Albain, Helen K Chew, Gary V Burton, Gordan Srkalovic, Bradley A McGregor, Lawrence E Flaherty, Danika L Lew, Julie R Gralow, Gabriel N Hortobagyi, Priyanka Sharma, Lajos Pusztai, George T Budd

**Affiliations:** Division of Hematology and Oncology, Cleveland Clinic Foundation, Taussig Cancer Institute, Cleveland, OH, United States; Department of Biostatistics, University of Washigton, SWOG Statistics and Data Management Center, Seattle, WA, United States; Division of Hematology and Oncology, Cleveland Clinic Foundation, Taussig Cancer Institute, Cleveland, OH, United States; Division of Hematology and Oncology, Mayo Clinic Comprehensive Cancer Center, Rochester, MN, United States; Division of Hematology and Oncology, Georgetown Lombardi Comprehensive Cancer Center, Washington, DC, United States; Division of Oncology, Allan Blair Cancer Center, Regina, Saskatchewan, Canada; Division of Hematology and Oncology, Loyola University Chicago, Stritch School of Medicine, Chicago, IL, United States; Division of Hematology and Oncology, University of California-Davis Comprehensive Cancer Center, Sacramento, CA, United States; Division of Hematology and Oncology, Louisiana State University Health Sciences Center, Shreveport, LA, United States; Division of Hematology and Oncology, University of Michigan Health - Sparrow, Herbert-Herman Cancer Center, Lansing, MI, United States; Dvision of Hematology and Oncology, Dana-Farber Cancer Institute, Boston, MA, United States; Division of Hematology and Oncology, Karmanos Cancer Institute/Wayne State University, Detroit, MI, United States; Department of Biostatistics, University of Washigton, SWOG Statistics and Data Management Center, Seattle, WA, United States; American Society of Clinical Oncology Association for Clinical Oncology, Alexandria, VA, United States; Division of Hematology and Oncology, University of Texas MD Anderson Cancer Center, Houston, TX, United States; Division of Hematology and Oncology, University of Kansas Medical Center, Kansas City, KS, United States; Division of Hematology and Oncology, Yale Cancer Center, New Haven, CT, United States; Division of Hematology and Oncology, Cleveland Clinic Foundation, Taussig Cancer Institute, Cleveland, OH, United States

## Abstract

**Background:**

S0221 investigated weekly vs every 2 weeks dosing of doxorubicin (A) and cyclophosphamide (C) followed by paclitaxel in patients with high-risk early breast cancer. After an interim analysis, random assignment to the 2 AC arms was stopped for futility, and the trial was modified to study only the paclitaxel schedules.

**Methods:**

Between December 2003 and November 2010, a total of 2716 patients were randomly assigned in a 2 × 2 factorial design to 15 weeks of weekly A and daily C vs 6 cycles of every 2 weeks AC; and weekly paclitaxel for 12 weeks vs 6 cycles of every 2 weeks paclitaxel. Between January 2011 and January 2012, an additional 578 patients were assigned to 4 cycles of every 2 weeks AC and randomly assigned to weekly vs every 2 weeks paclitaxel. Updated survival was assessed using log-rank tests and Cox regression models. We compared outcomes by breast cancer subtype as well.

**Results:**

At a median follow-up of 12.1 years, there were no statistically significant differences among the 4 treatment arms in disease-free survival (DFS) (*P *= .91) or overall survival (*P *= .34) in the original protocol. Among the 578 patients assigned AC for 4 cycles and randomly assigned to paclitaxel weekly vs every 2 weeks paclitaxel, there were no overall differences in DFS (*P *= .32) or overall survival (*P *= .42).

**Conclusion:**

As there were no statistically significant outcome differences in DFS or overall survival between the studied schedules of AC and paclitaxel with extended follow-up in the original or revised protocol, either paclitaxel schedule may be recommended, with selection based on toxicity, cost, or patient preference.

## Introduction

Even with the development of novel agents, cytotoxic chemotherapy remains a standard treatment for the majority of high-risk early stage breast cancer, and the magnitude of benefit depends on the risk of the cancer.^1^ Careful risk assessment of tumor biology can help right-size therapy decisions, and conventional clinicopathological factors associated with chemotherapy benefit include histological grade 3 tumor, high Ki-67 levels, low hormone receptor status, HER2 positivity or triple-negative status, and involvement of more than 3 lymph nodes.^2^ In hormone receptor–positive early breast cancer, additional genomic information can further select patients most likely to derive the greatest benefit from adjuvant chemotherapy.^3-5^ Anthracycline and taxane chemotherapy regimens remain the mainstay cytotoxic therapy routinely used in the adjuvant therapy of high-risk early breast cancer.^6^^,^^7^ There is preclinical and clinical evidence that increasing the dose-density of treatment by shortening the interval between treatment cycles (sequential or concurrent) from an every 3-week schedule to every 2 weeks,^8^ or to weekly administration, results in statistically significant improvement in disease-free survival (DFS), and the benefit was consistent despite the number of positive nodes, tumor size, menopausal status, and tumor estrogen receptor status.^9-11^ Some data have suggested that dose-dense chemotherapy does not increase adverse events and might be more effective in higher-risk patients with hormone receptor–negative tumors.^12^ Additionally, in the meta-analysis led by Early Breast Cancer Trialists’ Collaborative Group, the investigators showed that increasing dose density of chemotherapy drugs or delivering chemotherapy sequentially (rather than concurrently) reduced the 10-year breast cancer–related recurrence or death.^13^

S0221 was a previously reported phase III randomized trial performed by the North American Breast Cancer Intergroup (now known as the National Clinical Trials Network) investigating weekly doxorubicin (A) given with daily cyclophosphamide (C) plus granulocyte colony-stimulating factor (filgrastim or pegfilgrastim) for 15 weeks vs an every 2-week schedule AC for 6 cycles, followed by paclitaxel given every 2 weeks or weekly for 12 weeks as postoperative adjuvant therapy in node-positive or high-risk node-negative breast cancer.^14^ S0221 sought to compare alternative dose-dense regimens of adjuvant chemotherapy to assess the safety and efficacy of different dosing regimens.^14^

We previously reported the results of the original cohort of 2716 patients at 6 years of follow-up in those who were randomly assigned to the 2 AC arms and the 2 paclitaxel arms in a 2 × 2 factorial design. At 6-year follow-up, we reported no difference in DFS among the 4 treatment arms but statistically significant improvement in overall survival in the arm using every 2-weeks AC followed by every 2-weeks paclitaxel.^14^ There was also a nonsignificant trend toward improvement in DFS (*P *= .077) and overall survival (*P *= .067) in hormone receptor–negative, HER2-negative tumors.^14^ Here, we report the long-term outcomes, at 12.1 years, for this original cohort of 2716 patients and report, for the first time, the outcomes of a second cohort of 578 patients also treated as a part of S0221 according to the revised protocol who were assigned to treatment with 4 cycles of every 2 weeks AC but randomly assigned to either 12 weeks of therapy with paclitaxel weekly or every 2 weeks. Study data are being presented at the current 12.1-year mark to provide a long-term update of the original and revised protocol, including presentation of new as well as subgroup data that have not been previously presented.

## Methods

### Patients

Complete eligibility criteria have been previously reported.^14^ In brief, S0221 enrolled high-risk pathologic stage I-III breast cancer patients of all receptor subtypes. High risk was defined as node-positive (pN1-3), any primary tumor at least 2 cm, or any tumor at least 1 cm if it was hormone receptor negative or if it was hormone receptor positive with a 21-gene recurrence score of at least 26. Estrogen receptor and progesterone receptor positivity was defined as 10% or more estrogen receptor– or progesterone receptor–positive cells, and HER2 positivity was defined as 3 or higher on immunohistochemistry or amplification on fluorescence in situ hybridization. Receptor testing was performed locally and was not centrally confirmed. The study protocol was approved by the National Cancer Institute Central Institutional Review Board and the institutional review boards of each participating institution. All patients provided written informed consent for participation, and patient safety was monitored by an independent data safety monitoring committee.

### Study Procedures

The original study design was an open-label 2 × 2 factorial design with equal probability of receiving each treatment combination in an unstratified random assignment. The first factor compared A 60 mg/m^2^ intravenously (IV) day 1, C 600 mg/m^2^ IV day 1, and pegfilgrastim 6 mg subcutaneously on day 2, administered every 2 weeks for 6 cycles vs a continuous schedule of A 24 mg/m^2^ IV once per week, C 60 mg/m^2^ orally once per day, and filgrastim 5 µg/kg rounded to the nearer of 300 or 480 mg subcutaneously once per day, except for the days of IV drug administration, for 15 weeks. The second factor was subsequent paclitaxel 175 mg/m^2^ IV day 1 every 2 weeks with pegfilgrastim 6 mg subcutaneously day 2 repeated every 2 weeks for 6 cycles vs paclitaxel 80 mg/m^2^ IV once per week for 12 weeks. Trastuzumab therapy was recommended for HER2-positive patients following US Food and Drug Administration approval for adjuvant use on November 16, 2006, although whether it was administered was not tracked as part of the study. The amendment to allow trastuzumab use was distributed in November 2006 when 29.4% of patients had been enrolled.

A total of 2716 patients were randomly assigned from December 16, 2003, to November 9, 2010, to the original 2 × 2 design. At the time of the first interim analysis in September 2010, the observed hazard ratio (HR) for DFS for weekly AC with filgrastim vs AC every 2 weeks with pegfilgrastim was 1.21 (adjusting for the paclitaxel random assignment). The 99.5% confidence interval (CI) was 0.90 to 1.64, which suggested that it would be futile to continue random assignment to this schedule because it was unlikely that weekly AC would be superior to every 2 weeks. On the basis of the recommendation of the data safety monitoring committee, accrual was suspended in November 2010, and the trial reopened in December 2010 with all patients assigned to 4 cycles of AC administered every 2 weeks and randomly assigned only to the paclitaxel factor. A total of 578 additional patients were enrolled under this revised protocol design between January 21, 2011, and January 15, 2012.

### Statistical Methods

The primary outcome was DFS defined as time from registration to disease recurrence, new breast primary, or death due to any cause. This trial preceded the preceded the Standardized Definitions for Efficacy End Points (STEEP) criteria for adjuvant breast cancer clinical trial endpoints designed for consistent analysis, which recommended inclusion of nonbreast primaries as events in invasive DFS.^15^ Overall survival was a secondary outcome. Survival times were censored at last contact. Follow-up for survival was locked on January 15, 2024. The overall accrual goal was 3250. In the factorial design, each treatment factor was to be tested separately in a joint model with both factors (at 2-sided α = .05) if there was no statistically interaction of the 2 factors (α = .10). Power was 90% to test whether weekly administration was superior to dose-dense with a hazard ratio of 0.82 or less. Survival analysis methods included Kaplan–Meier plots, log-rank tests, and Cox regression analyses.

## Results

Efficacy

**Figure 1.**
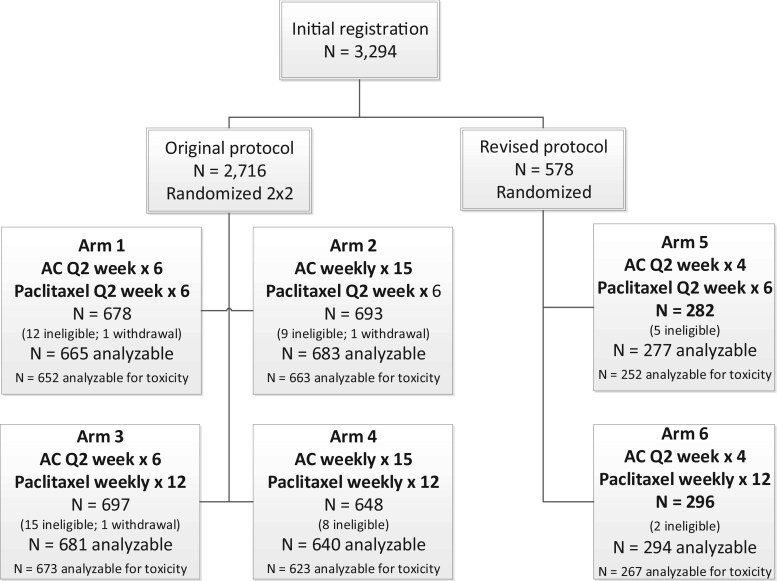
CONSORT diagram for the original and revised protocols of the SWOG S0221 trial. Abbreviations: AC = doxorubicin-cyclophosphamide; Q2 week = once every 2 weeks.

Figure 1 illustrates the trial design and number of randomly assigned patients in a CONSORT diagram from the original protocol of 2716 patients and the updated cohort on the revised protocol including 578 additional patients. Of those 578 patients, 282 (277 analyzable) were randomly assigned to the paclitaxel every 2 weeks arm, and 296 (294 analyzable) were randomly assigned to the paclitaxel weekly arm. The patient population under the revised protocol differed from that in the original protocol, as the patients tended to be older (median age = 53 vs 51 years), have less advanced disease (38% node-negative vs 23%), and to less frequently have hormone receptor–positive and HER2-negative disease (49% vs 56%) as shown in Table 1.

**Table 1.** Characteristics of patients entered on the original and revised protocols of S0221.

	Comparison of original revised protocols			Arms in revised protocol AC Q2W followed by:	
Baseline characteristics	Original protocol No. (%)	Revised Protocol No. (%)	*P*	Arm 5:^a^ Q2W x × 6 No. (%)	Arm 6:^b^ P weekly × 12 No. (%)
Patients					
Randomly assigned	2716	578		282	296
Ineligible, withdrew consent	47 (1.7)	7 (1.2)		5 (1.8)	2 (0.7)
Analyzed	2669	571		277	294
Age, median (range), y	51 (21- 86)	53 (23-79)	<.0001	52.7 (31 -79)	53.2 (23 -76)
Black race	304 (11)	75 (13)	.25	31 (11)	44 (15)
Male	18 (0.7)	5 (0.9)	.58	2 (0.7)	3 (1.0)
Menopausal status					
Missing data	36	10		5	5
Premenopausal	1251 (48)	314 (44)	.14	124 (46)	123 (43)
Postmenopausal	1382 (52)	314 (56)		148 (54)	166 (57)
Nodal status					
Missing data	9	1		1	0
Negative	620 (23)	214 (38)	<.0001	105 (38)	109 (37)
1-3 positive nodes	1047 (39)	201 (35)		103 (37)	98 (33)
≥4 positive nodes	993 (37)	155 (27)		68 (25)	87 (30)
Disease subtype					
Missing data	28	2		0	2
Hormone receptor positive/HER2 negative	1482 (56)	276 (49)	.004	129 (47)	147 (50)
HER2 positive	476 (18)	126 (22)		69 (25)	57 (20)
Hormone receptor negative/HER2 negative	683 (26)	167 (29)		79 (29)	88 (30)

Abbreviations: AC = doxorubicin and cyclophosphamide; P = paclitaxel; Q2W = every 2 weeks.

a AC Q2W × 4 → P Q2W × 6: Doxorubicin 60 mg/m^2^ intravenously day 1, cyclophosphamide 600 mg/m^2^ on day 1, pegfilgrastim 6 mg subcutaneously day 2, Q2W for 4 cycles followed by P 175 mg/m^2^ intravenously on day with 1 with pegfilgrastim 6 mg subcutaneously day 2 repeated Q2W for 6 cycles.

b Arm 6: AC Q2W × 4 → P weekly × 12: Doxorubicin 60 mg/m^2^ intravenously day 1, cyclophosphamide 600 mg/m^2^ on day 1, pegfilgrastim 6 mg subcutaneously day 2, Q2W for 4 cycles followed by P 80 mg/m^2^ given weekly for 12 weeks.

### Original Protocol

**Figure 2.**
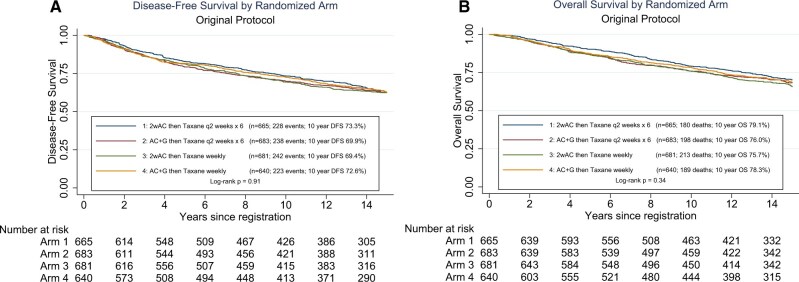
**A)** DFS of the 4 arms in the original protocol. **B)** OS as shown of the 4 arms in the original protocol. Abbreviations: AC = doxorubicin-cyclophosphamide; DFS = disease-free survival; OS = overall survival; q2 = every 2; G = Granulocyte Colony Stimulating Factor.

At the time of the primary analysis of the factorial design, an interaction between the 2 random assignments in the original protocol was found, necessitating analysis of all 4 arms separately. Now at a median follow-up of 12.1 years, there is no significant statistically difference in DFS between the 4 arms (*P *= .91) or in overall survival (*P *= .34) (Figure 2, A and B). Interestingly, arm 1 had the best overall survival among the 4 arms, though this is not statistically significant (*P *= .069). The interaction between the 2 random assignments was no longer significant (*P *= .53). There was also no difference in DFS if AC was given weekly or every 2 weeks (*P *= .86) or if paclitaxel was given weekly or every 2 weeks (*P *= .72) (Supplement S1: S1A, S1B) when tested in a 2 × 2 factorial design.

### Revised Protocol

There was no difference in DFS among the patients on the revised protocol in terms of the weekly or every 2 weeks paclitaxel schedule (*P *= .32) (Supplement S1: S1C). Treatment did not interact with disease subtype for DFS (*P *= .38). No treatment difference in overall survival (*P *= .42) was observed.

### Combined Analysis

**Figure 3.**
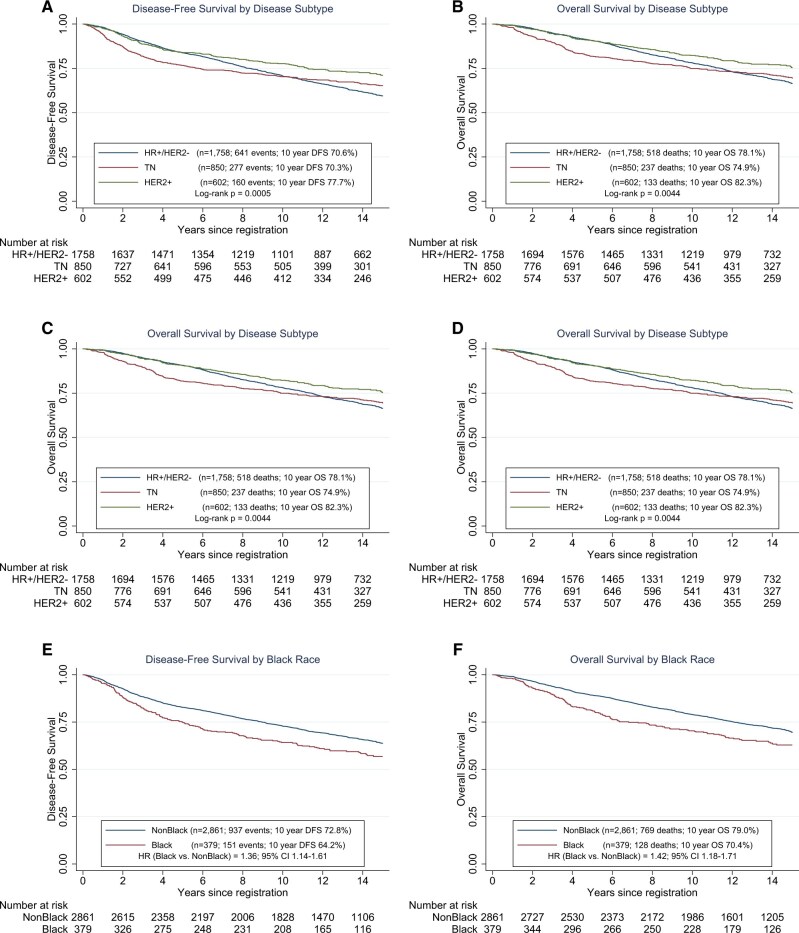
**A)** DFS of all patients stratified by receptor subtype. **B)** OS of all patients stratified by receptor subtype. **C)** DFS of all patients stratified by sex. **D)** OS of all patients stratified by sex. **E)** DFS of all patients stratified by race. **F)** OS of all patients stratified by race. Abbreviations: CI = confidence interval; DFS = disease-free survival; HR = hazard ratio; HR+ = hormone receptor positive; OS = overall survival; TN = triple-negative.

There were no significant statistically differences in efficacy outcomes based on the dosing schedule (Table S1). There were also no significant differences by treatment arm when stratified by disease subtype. When patients were compared across disease subtypes without adjustment for pretreatment prognostic variables, the HER2-positive cohort had the highest 10-year DFS (77.7%) compared with hormone receptor–positive and HER2-negative (70.6%) or hormone receptor–negative/HER2-negative (70.3%) cohorts (*P*  = .0005) (Figure 3, A). The overall survival in the HER2-positive cohort had a 10-year overall survival of 82.3% compared with hormone receptor–positive and HER2-negative (78.1%) or hormone receptor–negative/HER2-negative (74.9%) cohorts (*P *= .0044) (Figure 3, B). Cox regression of DFS and overall survival adjusting for number of positive nodes (0, 1-3, ≥4) gives the same pattern of results, but the proportional hazards assumption fails as may be deduced from the Kaplan–Meier graphs. When stratified by hormone receptor status and HER2 status, DFS and overall survival were initially the highest in hormone receptor–positive/HER2-positive patients, but the survival curves came together with long-term follow-up. Among the 602 patients with HER2-positive disease, 227 were hormone receptor negative and 375 were hormone receptor positive. The hormone receptor positive patients had better DFS (Supplement S2: S2A) and overall survival (Supplement S2: S2B) in the first 5 years (*P *= .026) than hormone receptor–negative patients. However, the DFS (Supplement S2: S2C) and overall survival (Supplement S2: S2D) curves come together with longer follow-up resulting in no significant difference by hormone receptor status among HER2 positive patients.

We also stratified the entire cohort based on sex though only 23 men were enrolled. Although women have superior DFS and overall survival compared with men, because of the small number of men and wide confidence interval, these data should be interpreted with caution (Figure 3, C and D). We also stratified patient population based on race with 379 Black patients enrolled compared with 2861 non-Black patients. Black patients had worse DFS and overall survival (DFS: HR = 1.36, 95% CI = 1.14 to 1.61; 10-year DFS: 64.2% vs 72.8%; overall survival: HR = 1.42, 95% CI = 1.18 to 1.71; 10-year overall survival: 70.4% vs 79.0%) (Figure 3, E and F). However, Black patients were more likely to have hormone receptor–negative/HER2-negative disease (36.9% [139 of 377]) and less likely to have hormone receptor–positive/HER2-negative disease (41.1% [155 of 377]) compared with the distribution in non-Black patients (hormone receptor negative/HER2 negative: 25.1% [711 of 2833]; hormone receptor positive/HER2 negative: 56.6% [1603 of 2833]). Considering only the first 5 years, we compared Black patients with non-Black patients separately by subtype. For hormone receptor–negative/HER2-negative disease, there was no difference in DFS between Black patients and non-Black patients (HR = 0.96, 95% CI = 0.66 to 1.41). For hormone receptor–positive/HER2-negative disease, Black patients had much higher event rates (HR = 2.01, 95% CI = 1.45 to 2.81; *P *< .001). Similarly for HER2-positive disease, Black patients had higher event rates (HR = 1.74, 95% CI = 1.06 to 2.85; *P *= .027).

### Safety

Table 2 shows grade 3 or higher adverse events from all 6 arms: arms 1-4 of the original protocol and arms 5-6 of the revised protocol. Arms 5-6, where AC was administered for 4 cycles every 2 weeks, had lower grade 3 or higher hematological toxicity compared with arms 1-4. In particular, grade 3 or higher neutropenia was up to 6.7% vs up to 26.4%, grade 3 or higher anemia up to 3.2% vs up to 10.5%, and grade 3 or higher thrombocytopenia up to 0.4% vs up to 3.0%. Grade 3 or higher cardiac events (which included arrhythmias or general cardiac dysfunction) were the lowest in the weekly AC arms (arms 2 and 4: up to 0.6%), followed by every 2 weeks AC × 4 cycles (arms 5 and 6: up to 2.2%), and were the highest in the every 2 weeks AC × 6 cycles (arms 1 and 3: up to 3.6%). There was more skin toxicity with weekly AC (up to 16.1%) compared with the other 4 arms (up to 4.0%) and notably more hand-foot syndrome up to 14.4% (arms 2 and 4, respectively) compared with the other 4 arms ranging up to 2.1%. Arms 2 and 4 also had more gastrointestinal toxicity and grades 3 or higher events were as high as 18.8%. There was more neuropathy in arms (up to 19.0% vs up to 10.5%) where paclitaxel was given every 2 weeks × 6 cycles (arms 1, 2, and 5). There were no differences in risk of pulmonary toxicity or secondary malignancy in any of the 6 arms, and the incidence of the latter remained low (<1%).

**Table 2.** CTCAE grade 3 or higher toxicity in arms 1-6.

Adverse event	CTCAE toxicity grade ≥ 3 No. (%)					
	Arm 1^a^ AC Q2W × 6 → P Q2W × 6 (*n* = 652)	Arm 2^b^ AC weekly × 15 → P Q2W × 6 (*n* = 663)	Arm 3^c^ AC Q2W × 6 → P weekly × 12 (*n* = 673)	Arm 4^d^ AC weekly × 15→ P weekly × 12 (*n* = 623)	Arm 5^e^ AC Q2W × 4 → P Q2W × 6 (*n* = 252)	Arm 6^f^ AC Q2W × 4 → P weekly × 12 (*n* = 267)
Neutropenia	167 (25.6)	149 (22.5)	178 (26.4)	151 (24.2)	5 (2.0)	18 (6.7)
Anemia	59 (9.0)	35 (5.3)	71 (10.5)	34 (5.5)	8 (3.2)	6 (2.2)
Thrombocytopenia	16 (2.5)	20 (3.0)	19 (2.8)	19 (3.0)	1 (0.4)	0 (0)
Cardiac^g^	21 (3.2)	4 (0.6)	20 (3.0)	3 (0.5)	3 (1.2)	6 (2.2)
Constitutional symptoms	66 (10.1)	55 (8.3)	77 (11.4)	55 (8.8)	21 (8.3)	15 (5.6)
Dermatology	26 (4.0)	107 (16.1)	22 (3.3)	98 (15.7)	8 (3.2)	7 (2.6)
Hand-foot syndrome	14 (2.1)	91 (13.7)	10 (1.5)	90 (14.4)	3 (1.2)	2 (0.7)
Gastrointestinal	76 (11.7)	116 (17.5)	96 (14.3)	117 (18.8)	22 (8.7)	25 (9.4)
Infection	67 (10.3)	31 (4.7)	67 (10.0)	45 (7.2)	20 (7.9)	29 (10.9)
Neuropathy^h^	100 (15.3)	101 (15.2)	71 (10.5)	54 (8.7)	48 (19.0)	28 (10.5)
Pain	71 (10.9)	91 (13.7)	35 (5.2)	40 (6.4)	36 (14.3)	12 (4.5)
Pulmonary	17 (2.6)	21 (3.2)	19 (2.8)	13 (2.1)	6 (2.4)	7 (2.6)
Secondary malignancy	2 (0.3)	2 (0.3)	4 (0.6)	3 (0.5)	1 (0.4)	0 (0)

Abbreviations: AC = doxorubicin and cyclophosphamide; CTCAE = Common Terminology Criteria for Adverse Events; P = paclitaxel; Q2W = every 2 weeks.

a Arm 1: AC Q2W × 6 → P Q2W × 6: Doxorubicin 60 mg/m^2^ intravenously day 1, cyclophosphamide 600 mg/m^2^ on day 1, pegfilgrastim 6 mg subcutaneously day 2, Q2W for 6 cycles followed by P 175 mg/m^2^ intravenously on day with 1 with pegfilgrastim 6 mg subcutaneously day 2 repeated Q2W for 6 cycles.

b Arm 2: AC weekly × 15 → P Q2W × 6: Doxorubicin 24 mg/m^2^ intravenously once per week, cyclophosphamide 60 mg/m^2^ orally once per day, and filgrastim 5 µg/kg rounded to the nearest 300 or 480 mg given subcutaneously once per day except for the days of intravenous administration for 15 weeks followed by P 175 mg/m^2^ intravenously on day with 1 with pegfilgrastim 6 mg subcutaneously day 2 repeated every 2 weeks for 6 cycles.

c Arm 3: AC Q2W × 6 → P weekly × 12: Doxorubicin 60 mg/m^2^ intravenously day 1, cyclophosphamide 600 mg/m^2^ on day 1, pegfilgrastim 6 mg subcutaneously day 2, every 2 weeks for 6 cycles followed by P 80 mg/m^2^ given weekly for 12 weeks.

d Arm 4: AC weekly × 15→ P weekly × 12: Doxorubicin 24 mg/m^2^ intravenously once per week, cyclophosphamide 60 mg/m^2^ orally once per day, and filgrastim 5 µg/kg rounded to the nearest 300 or 480 mg given subcutaneously once per day except for the days of intravenous administration for 15 weeks followed by P 80 mg/m^2^ given weekly for 12 weeks.

e Arm 5: AC Q2W × 4 → P Q2W × 6: Doxorubicin 60 mg/m^2^ intravenously day 1, cyclophosphamide 600 mg/m^2^ on day 1, pegfilgrastim 6 mg subcutaneously day 2, Q2W for 4 cycles followed by P 175 mg/m^2^ intravenously on day with 1 with pegfilgrastim 6 mg subcutaneously day 2 repeated Q2W for 6 cycles.

f Arm 6: AC Q2W × 4 → P weekly × 12: Doxorubicin 60 mg/m^2^ intravenously day 1, cyclophosphamide 600 mg/m^2^ on day 1, pegfilgrastim 6 mg subcutaneously day 2, Q2W for 4 cycles followed by P 80 mg/m^2^ given weekly for 12 weeks.

a Cardiac includes arrhythmia and general cardiac adverse events.

b Neuropathy includes sensory and motor neuropathy.

## Discussion

Dose-dense chemotherapy, at least for anthracycline and taxane-based regimens, appears superior to less intense schedules, as predicted by mathematical modeling.^9^^,^^16^ S0221 sought to compare alternative dose-dense schedules to inform standard therapy recommendations and future trial design. Based on findings of S0221, there were no significant statistically differences among the 4 treatment arms in terms of DFS (*P *= .91) or overall survival (*P *= .34) in the original protocol or DFS (*P *= .32) or overall survival (*P *= .42) in the revised protocol, but the weekly regimen did lead to more treatment-related skin toxicity and a higher incidence of grade 3 or higher hand-foot syndrome. Overall, dose-dense paclitaxel administered either weekly or every 2 weeks provides similar outcomes, allowing the choice of regimen to be based on toxicity or patient preference. It should be noted that the toxicity of 4 cycles of every 2 weeks paclitaxel is less than the 6 cycles that were administered in S0221. Although the arms in S0221 were not designed to compare the efficacy of AC × 4 vs 6, equivalent efficacy and more toxicity with 6 cycles has also been previously reported.^17^ We note that patients in the revised protocol had overall improved outcomes than those entered on the original protocol (Figure 2), although this difference was not statistically significant after adjustment for nodal status (*P *= .54) and could be accounted for by differences in the baseline demographics and tumor characteristics of the newer cohort (ie, older median age, fewer premenopausal patients, more patients with node negative disease, and fewer patients with 4 or more lymph nodes) (Table 1).

At the time of initial analysis, a signal for improved outcome as measured by numerical improvement in DFS or overall survival in patients with hormone receptor–negative and HER2-negative tumors was seen in arm 1 (where AC and paclitaxel were given every 2 weeks).^14^ It is worth noting that this was an unplanned subset analysis after the discovery of an unexpected interaction between the 2 random assignment schemas in the original protocol, and we cannot safely draw any efficacy conclusions particularly given the lack of benefit toward any treatment schema at this final analysis at 12.1 years.

Given the differences in biology and natural history between breast cancer subtypes, it would not be unexpected to find differences in outcome by treatment schedule between subtypes. With long-term follow-up, differences in the treated natural histories of the histologic subtypes are noted. Patients with HER2-positve disease had the most favorable outcome, and these patients had a statistically superior DFS and overall survival at 12.1 years compared with hormone receptor–positive and HER2-negative and hormone receptor–negative/HER2-negative cohorts.

A likely explanation for the superior DFS and overall survival in HER2-positive cohort is the use of trastuzumab for many of those patients following Food and Drug Administration approval of trastuzumab for early breast cancer in 2006 and a protocol amendment encouraging its use, though the use was not tracked as part of the study. It is possible that addition of trastuzumab alone could have contributed to a significant statistically significant improvement in risk of recurrence or breast cancer–related mortality.^18^ We acknowledge that although better efficacy outcomes in a HER2-positive cohort treated with trastuzumab is consistent with epidemiologic data,^19^^,^^20^ this study is unique in its reporting of relatively long-term outcomes of a heterogenous high-risk early breast cancer cohort. One limitation of the study is a lack of clarity regarding use of trastuzumab in the HER2-positive cohort. Although treating physicians had to provide documentation on whether they intended to use trastuzumab, data on which patients actually received trastuzumab were not collected. In terms of subgroup analyses, the study did provide some hypothesis-generating trends. Notably, all males had hormone receptor breast cancer with 2 also being HER2 positive. The finding that survival was worse in Black women and especially in hormone receptor–positive disease has been shown before in the literature. A meta-analysis by Torres et al.^21^ showed that the Black population had poorer survival for all subtypes but was considerably elevated in hormone receptor–positive and HER2-negative cancers.

During the first 10 years of follow-up, patients with hormone receptor–positive and HER2-negative tumors had the best DFS, but after the first 10 years, the continual trend for late relapse of hormone receptor–positive/HER2-negative disease resulted in a worse long-term outcome for patients with hormone receptor–positive/HER2-negative tumors in this high-risk cohort. Information regarding extended adjuvant therapy for these patients was not collected, so these data provide no insight into the value of extended endocrine therapy. In terms of toxicity, we note that patients in arms 5 and 6 had the least amount of bone marrow dysfunction likely owing to a younger age of this population and representing a patient cohort with a lower overall anthracycline dose (4 cycles, instead of 6 cycles).

The final analysis of S0221 showed no difference in efficacy in any of the 4 treatment arms of anthracycline and/or taxane schedule or in the 2 arms of the revised protocol. Among the breast cancer subtypes, patients with HER2-positive tumors had the best DFS and overall survival compared with patients with other subtypes in the final combined analysis, although it is unclear if that benefit is driven by anthracycline benefit or use of trastuzumab-containing regimens. Weekly anthracycline for 15 weeks did not improve efficacy but did add notable toxicity. The selection and schedule of regimens should depend on the individual risk of breast cancer and patient preferences while keeping the toxicity potential in mind.

## Supplementary Material

pkag024_Supplementary_Data

## Data Availability

Data are the property of SWOG and participating sites and can be made available upon request at the discretion of SWOG data management.
