# Supplementary material for: Long-term follow-up of S0221, comparing alternative dose-schedules of anthracycline and taxane therapy in early breast cancer
Source: JNCI Cancer Spectr. 2026 Mar 15;10(2):pkag024. doi: 10.1093/jncics/pkag024 (PMC13102176; doi:10.1093/jncics/pkag024)
Supplement: pkag024_Supplementary_Data [file pkag024_supplementary_data.docx]

Supplementary Appendix

Table S1: Statistical testing and modeling of DFS and OS by treatment assignment and protocol version

Figure S1: S1A: Disease-free survival stratified by Q2W vs. weekly AC schedule in the original protocol

Figure S1: S1B: Disease-free survival stratified by Q2W vs. weekly P schedule in the original protocol

Figure S1: S1C: Disease-free survival stratified by Q2W vs. weekly P schedule in the revised protocol

Figure S2: S2A: Disease-free survival stratified by HR-status and HER2-status

Figure S2: S2B: Overall survival stratified by HR-status and HER2-status

Figure S2: S2C: Disease-free survival by HR status in HER2-positive patients

Figure S2: S2D: Overall survival by HR status in HER2-positive patients

| Comparison | Hazard ratio | 95% CI | p-value |
| --- | --- | --- | --- |
| DFS Original protocol (4 arms) | | | |
| Log-rank p-value (comparing 4 arms) |  |  | 0.91 |
| Log-rank p-value stratified by receptor status |  |  | 0.94 |
| Arm 2 vs. Arm 1 | 1.05 | 0.88 - 1.26 | 0.57 |
| Arm 3 vs. Arm 1 | 1.07 | 0.89 - 1.28 | 0.49 |
| Arm 4 vs. Arm 1 | 1.04 | 0.86 - 1.25 | 0.71 |
|  |  |  |  |
| DFS (original protocol 2 factors) |  |  |  |
| AC weekly (Arms 2 & 4) vs. AC Q2W (Arms 1 & 3) | 1.01 | 0.89 - 1.15 | 0.86 |
| P weekly (Arms 3 & 4) vs. P Q2W (Arms 1 & 2) | 1.02 | 0.90 - 1.16 | 0.72 |
|  |  |  |  |
| OS original protocol (4 arms) | | | |
| Log-rank p-value (comparing 4 arms) |  |  | 0.34 |
| Log-rank p-value stratified by receptor status |  |  | 0.44 |
| Arm 2 vs. Arm 1 | 1.12 | 0.91 - 1.37 | 0.29 |
| Arm 3 vs. Arm 1 | 1.20 | 0.99 - 1.47 | 0.07 |
| Arm 4 vs. Arm 1 | 1.12 | 0.91 - 1.37 | 0.28 |
|  |  |  |  |
| OS original protocol (2 factors) |  |  |  |
| AC weekly (Arms 2 & 4) vs.   AC Q2W (Arms 1 & 3) | 1.02 | 0.88 - 1.17 | 0.83 |
| P weekly (Arms 3 & 4) vs.  P Q2W (Arms 1 & 3) | 1.10 | 0.95 - 1.26 | 0.19 |
|  |  |  |  |
| DFS revised protocol (2 arms) |  |  |  |
| Log-rank p-value |  |  | 0.32 |
| P weekly (Arm 6) vs P Q2W (Arm 5) | 0.85 | 0.62 - 1.17 | 0.32 |
|  |  |  |  |
| OS revised protocol (2 arms) |  |  |  |
| Log-rank p-value |  |  | 0.42 |
| P weekly (Arm 6) vs. P Q2W (Arm 5) | 0.86 | 0.60 - 1.24 | 0.42 |

Table S1

Figure S1: S1A

Figure S1: S1B

Figure S1: S1C

Figure S2: S2A

Figure S2: S2B

Figure S2: S2C

Figure S2: S2D
